# Continuity and discontinuity of care among older patients in Danish general practice: a retrospective cohort study

**DOI:** 10.3399/BJGPO.2023.0081

**Published:** 2023-09-06

**Authors:** Jonas K Olsen, Troels Kristensen

**Affiliations:** 1 Research Unit for General Practice, Institute of Public Health, University of Southern Denmark, Odense, Denmark; 2 Danish Centre for Health Economics, University of Southern Denmark, Odense, Denmark

**Keywords:** general practice, primary health care, family practice, aged, physician–patient relations, continuity of patient care, provider shifts, cohort study, longitudinal, site-level continuity of care

## Abstract

**Background:**

Continuity of care (COC) for older adults has been associated with lower use of healthcare services, decreased risk of hospitalisation, and lower mortality. However, research on COC in older adults is limited by short time periods and small sample sizes. Long-term COC can only develop if the patient stays with the general practice for ≥10 years. Therefore, research that focuses on long duration and broader populations is needed.

**Aim:**

To measure the extent of longitudinal site-level COC in general practice and listing duration of the patient–general practice relation for all older Danish citizens.

**Design & setting:**

Retrospective cohort study of all patients aged ≥65 years on 31 December 2021 listed with a Danish general practice (*N* = 1 144 941 persons).

**Method:**

Individual-level register data were used on start and end dates for listing with a general practice to analyse site-level COC by number of changes and listing duration of the patient–general practice relation from January 2007–December 2021.

**Results:**

During the 15 years, 39.3% of older adults did not change general practice. Among the remaining 60.7%, who experienced discontinuity of care, 34.0% changed once, 16.3% changed twice, and 6.3% changed three times. Overall, <5% changed general practice >3 times. The duration of the patient–general practice relations were on average 9.5 years. Overall, 27.5% lasted 0–4 years, 33.7% lasted 5–9 years, and 38.8% lasted ≥10 years.

**Conclusion:**

Danish general practice provides high levels of site-level COC for their older patients. On average, patients aged ≥65 years changed general practice once and had a patient–general practice relation length of 9.5 years.

## How this fits in

Older adults benefit from COC. It has been found to lower the use of healthcare services, as well as decrease the risk of hospitalisation and mortality. However, studies on COC have been limited by short time periods or small populations. This long-term nationwide study of general practice COC focused on dis(continuity) and listing duration of the patient–general practice relation for all Danish citizens aged ≥65 years.

## Introduction

COC for older adults in general practice has been associated with lower use of healthcare services, risk of hospitalisation, and mortality.^1–3^ Thus, circumstances where older patients rarely change general practice may reflect higher quality of care. Studies of COC for older patients is especially important owing to their high levels of multimorbidity,^4^ hospitalisations,^1^ polypharmacy,^5^ and use of healthcare services.^6,7^ However, previous literature on COC in older adults is limited by short time periods of 3–5 years or by small samples of a few thousand.^8^

One of the challenges with a short analysis period is that it does not allow for measurement and analysis of long-term COC. Long-term COC has no absolute definition in length but ≥10 years has been used to describe it.^9^ Studies of long-term COC may be required to measure potential effects of COC that may take several years to develop. One such case may be the named accountable GP programme in the UK from 2014. After 9 months no effect was seen in number of GP contacts, referrals, or use of blood pressure or HbA1c diagnostic tests.^10^ After 2 years, again, no improvement was seen in unplanned hospitalisations.^11^

COC has been measured in numerous ways and there is no consensus on a single preferable measure.^12^ The majority of the literature on COC focus on the patient–physician relation, where continuity can only be provided by a single physician.^12^ However, several primary care list systems are based on general practices with multiple GPs or healthcare centres comprising a joint team of health professionals, including doctors, nurses, physiotherapists, and other employees, as the main providers of continuity for the patient rather than the individual GP.^3,13–16^ Indicating that COC might be more accurately assessed at the site-level compared with the physician-level in these list systems. The Danish listing system ensures all citizens a general practice or healthcare centre, which, in turn for a capitation fee and fee-for-service, must provide healthcare services and act as gatekeeper to most specialist care.^14^ Furthermore, the listing system allows for measuring site-level COC in terms of the number of changes and length of time the patient has been listed in the specific general practice.

This article aimed to measure the extent of longitudinal site-level COC in general practice and listing duration of the patient–general practice relation for all older Danish citizens.

## Method

### Study design

A nationwide register-based cohort study was undertaken in Denmark. It assessed site-level COC in general practice in terms of number of general practice changes per patient and listing duration of patient–general practice relation for all Danish citizens aged ≥65 years.

### Setting

Denmark is a Scandinavian country with 5.8 million citizens. In Denmark, family medicine is a specialty in line with other medical specialties. To become a GP requires authorisation from the Danish Board of Health, which is based on 5 years of specialist training and courses. The healthcare system is tax-funded and virtually free of charge at the level of service. More than 98% of the population are listed with a specific general practice represented by a provider number. Danish GPs are paid capitation fees (1/3) based on the listing of patients and fees for services (2/3).^14^ Patients are free to choose a general practice if their list is open. General practices can choose to close their list if they have 1600 patients listed. Whether the shift from one general practice to another is free of charge or not depends on the circumstances. Change of general practice is free if the patient changes address, when turning 15 years of age, and if the current general practice closes, changes owner, or is subdivided. Otherwise, patients must pay a fee of approximately 29 Euros (approximately 25 GBP) as of 2022 to change general practice. Some general practices share their patient list across GPs (partnership practices) whereas others do not (cooperative and solo practices). The number of practices not sharing their patient list among GPs are declining, with 41% not sharing the patient list.^17^ Partnership practices are in general staffed by 2–5 GPs.^18^

### Study population and data collection

All data were provided by the Danish regions. The study population included all persons aged ≥65 years who had been enrolled with ≥1 Danish general practices between 1 January 2007 and 31 December 2021. They were included if they were still enrolled with a general practice and alive at the end of the study period.

### Statistical methods

Number of changes between general practice and the duration of enrolments were measured per patient during the study period (January 2007–December 2021). Length of patient–general practice enrolment was calculated as time listed in each general practice. All patient characteristics are described with a mean, standard deviation (SD), coefficient of variation, minimum, maximum, and fifth (p5) and 95^th^ (p95) percentiles. All analyses were performed using Stata (version 17.0).

## Results

### Patient characteristics

The patient characteristics in Table 1 show the 1 144 941 Danish citizens aged ≥65 years who were listed with a general practice and alive at the end of the 15-year study period ultimo 2021. Females comprised 54.0% of the group of all older patients. The average age of the listed population of older patients was 75.3 years, with 66.0 years (p5) and 88.6 years (p95). The average number of changes between general practices was one during the 15-year period, with a variation of 1.1 (SD) with 0 (p5) and 3 (p95). On average, the patient–general practice relation duration length was 9.5 years with 3.8 (p5) and 15.0 (p95).

**Table 1.** Patient characteristics of the Danish general practice patient population aged ≥65 years between January 2007 and December 2021

Category	*n* (%)	Mean	Standard deviation	Coefficient of variation	Min	p5	p95	Max
Total	1 144 941	—	—	—	—	—	—	—
Female	618 333 (54.0)	—	0.5	0.9	—	—	—	—
Male	526 608 (46.0)	—	0.5	1.1	—	—	—	—
Age in 2021, years	1 144 941	75.3	7.0	0.1	65.0	66.0	88.6	107.0^a^
Changes of general practice	1 144 941	1.0	1.1	1.1	0	0	3	38
Mean relation duration, days	1 144 941	3476	1666	0	0	1369	5478	5478
Mean relation duration, years	1 144 941	9.5	4.6	0.5	0.0	3.8	15.0	15.0
Change of health insurance group	1 144 941	0.04	0.3	7.0	0.0	0.0	0.0	21.0
Unique general practices per patient	1 144 941	2.0	1.1	0.6	1.0	1.0	4.0	28.0

^a^Age was capped at 107 years to ensure patient anonymity. p5 = fifth percentile. p95 = 95^th^ percentile.

Table 2 displays the number of general practice changes across age groups. No change of general practice was seen for 39.3% of the population of older Danish citizens. The majority of patients (60.7%) changed general practice at least once. Among the patients with discontinuity, 34.0% had one change, 16.3% had two changes, 6.3% had three changes, and a fraction of <5% had ≥4 changes during the 15 years.

**Table 2.** Number and proportions of general practice changes across age groups among Danish citizens aged ≥65 years between January 2007 and December 2021

	General practice changes, *n* (%)									
Age band, years	0	1	2	3	4	5	6	7	≥8	Total
65–69	122 260 (38.6)	105 101 (33.2)	52 695 (16.6)	21 235 (6.7)	9026 (2.8)	3503 (1.1)	1472 (0.5)	691 (0.2)	826 (0.3)	316 809 (27.7)
70–74	124 478 (40.2)	103 006 (33.3)	49 900 (16.1)	19 455 (6.3)	7797 (2.5)	2862 (0.9)	1140 (0.4)	440 (0.1)	473 (0.2)	309 551 (27.0)
75–79	101 973 (39.9)	88 244 (34.6)	40 559 (15.9)	15 310 (6.0)	5750 (2.3)	2194 (0.9)	751 (0.3)	299 (0.1)	272 (0.1)	255 352 (22.3)
80–84	59 712 (40.0)	52 145 (35.0)	23 274 (15.6)	8813 (5.9)	3384 (2.3)	1122 (0.8)	391 (0.3)	152 (0.1)	141 (0.1)	149 134 (13.0)
85–89	28 691 (37.9)	26 843 (35.4)	12 703 (16.8)	4740 (6.3)	1822 (2.4)	589 (0.8)	213 (0.3)	75 (0.1)	65 (0.1)	75 741 (6.6)
≥90	13 166 (34.3)	13 757 (35.9)	7035 (18.3)	2871 (7.5)	983 (2.6)	362 (0.9)	115 (0.3)	32 (0.1)	33 (0.1)	38 354 (3.3)
Total	450 280 (39.3)	389 096 (34.0)	186 166 (16.3)	72 424 (6.3)	28 762 (2.5)	10 632 (0.9)	4082 (0.4)	1689 (0.1)	1810 (0.2)	1 144 941 (100)

All percentages are row percentages except last column, which is column percentages.

### Number of general practice changes

The largest of the age groups was the group aged 65–69 years, which comprised 27.7% of the older Danish patients (Table 2). This proportion decreased to 3.3% for the group of ≥90 years. Despite the decrease in the absolute number of older patients in the higher age groups, the proportions of the number of general practice changes remained stable across age groups, that is, the oldest old or youngest old citizens did not have remarkably more or fewer changes than the remaining groups.

### Patient–general practice relation

Table 3 shows the duration of the patient–general practice relation across age groups. Of all older citizens, 27.5% had a patient–general practice relation of 0–4 years. A patient–general practice relation of ≥10 years was most common (38.8%), and 33.7% had been listed with the same general practice for 5–9 years. Thus, in total 72.5% had been enrolled with a general practice for ≥5 years. There was no remarkable variation in the length of the patient–general practice relation across the applied age groups.

**Table 3.** Patient–general practice relation duration versus age bands among Danish citizens aged ≥65 years between January 2007 and December 2021

	Patient–general practice relation duration, years, *n* (%)			
Age, years	0–4	5–9	≥10	Total
65–69	92 691 (29.3)	104 180 (32.9)	119 938 (37.9)	316 809 (27.7)
70–74	84 775 (27.4)	102 112 (33.0)	122 664 (39.6)	309 551 (27.0)
75–79	67 150 (26.3)	87 582 (34.3)	100 620 (39.4)	255 352 (22.3)
80–84	38 290 (25.7)	51 702 (34.7)	59 142 (39.7)	149 134 (13.0)
85–89	20 612 (27.2)	26 644 (35.2)	28 485 (37.6)	75 741 (6.6)
≥90	11 602 (30.2)	13 703 (35.7)	13 049 (34.0)	38 354 (3.3)
Total	315 120 (27.5)	385 923 (33.7)	443 898 (38.8)	1 144 941 (100)

All percentages are row percentages except last column, which is column percentages.

## Discussion

### Summary

On average, older Danish patients changed general practice once during the 15 years of January 2007–December 2021. Most common was zero changes (39.3%), followed by discontinuity in terms of one (34.0%), two (16.3%), and three (6.3%) changes. Overall, <5% changed general practice >3 times. There was no clinically significant difference in the distribution of general practice changes across age groups. The relations between the patient and the general practice were long, with 72.5% lasting >5 years. The durations were as follows: 27.5% lasted 0–4 years; 33.7% lasted 5–9 years; and 38.8% lasted ≥10 years.

### Strengths and limitations

To the authors' knowledge, this is the first long-term nationwide study of general practice COC in older adults focusing on both (dis)continuity and specific listing duration. A strength of the study was the use of individual-level comprehensive detailed longitudinal and virtually complete nationwide population register data for all citizens who were listed in a Danish general practice within the past 15 years. A feature that allowed all the patient–general practice relations to be analysed and limited the risk of selection bias.

A fundamental problem in research about COC is choosing the operationalisation of the concept and level at which to measure COC. Here the actual link has been used from the administrative registration of patients rather than an algorithm linking patient and general practices.^19^ Choosing the person-level for measurement risks underestimating COC if the site provides continuity as a healthcare team, whereas choosing the site-level risks overestimating continuity if the healthcare professionals at the site do not collaborate in patient care. Site-level COC was chosen to be used, which is relevant in general practice listing systems, such as the Danish, and enabled longitudinal COC by not being depended on a single provider who may have changed practice or retired. The latter have been a concern in Denmark and other countries,^20,21^ with 8.9% of general practice owners being aged ≥65 years as of 2022 in Denmark.^17^ Furthermore, site-level COC allows for informational continuity by each team member having access to the patient’s medical history. In addition, site-level COC has been associated with higher levels of preventive services independently of physician-level COC.^22,23^ These characteristics confirm that site-level COC is a feasible measure with high generalisability to use in large-scale population-based studies. Finally, measurement of site-level COC allows researchers and policymakers to identify areas where COC might be lacking and develop targeted interventions to improve COC.

A weakness of the applied real-world register data for this study is that GPs can choose to consolidate into a partnership practice. Under these circumstances, sometimes patients in a general practice are transferred to a single provider number even if multiple provider numbers contributed patients to the GP partnership. Under these special circumstances patients would be counted as having changed GP even though they may still be seen by the same provider and healthcare professionals. During the study period, the number of general practices sharing their patient list increased from 805 practices constituting 37.3% of all general practices to 980 practices constituting 58.5% of all practices.^18^ Therefore, this limitation of the registrations may have contributed to overestimate the number of actual general practice changes.

Another weakness is when a GP takes over a provider number from another GP then the provider number may stay the same. This is often the case in partnership practices with more GPs. Only when a single-handed GP retires and a new GP takes over will a new provider number be assigned. However, no matter what kind of change there is within a general practice, the patients are notified of the change of GP and are presented with the opportunity of changing general practice free of charge. Finally, the study only included patients who were alive ultimo December of 2021. Patients emigrating, dying, or otherwise leaving the listing system in Danish general practice during the period of January 2007–December 2021 were excluded. However, patients returning or immigrating to Denmark or otherwise joining the listing system during the analysis period were included. These characteristics of the available data and applied method may have contributed to skew the measured continuity in terms of changes and relation lengths towards shorter durations.

### Comparison with existing literature

To the authors’ knowledge, only a few studies have reported on frequencies of change of general practice or length of the patient–general practice relation for the older population.^9,24,25^

Different from the results in the present study, two North American studies, one from 1996 and one from 2005, found that Medicare beneficiaries aged 65–75 years and adults in the rural Southeastern US most often had ties to a physician for <5 years.^9,24^ In contrast, another North American study from 2004 found longer listing durations, similar to the present study, with a mean and median length of 10.3 and 8.0 years, respectively.^25^ The later study asked patients to report relation length in a questionnaire allowing for durations >15 years, whereas the present study had a maximum duration of 15 years. A possible reason for the shorter length of ties in the first two studies may be the focus on ties to a specific physician rather than a general practice. In line with this difference in unit of analysis, a Dutch study from 2016, focusing on patient–GP COC, found that 43% of patients aged ≥65 years had the same GP for at least 6 years, whereas the present study found that a larger percentage (72.5%) had the same general practice for at least 5 years. In Denmark, the number of solo practices is declining and many former solo practices are entering collaboratives,^26^ thus extending the relevance of investigating the effect of site-level COC.

The present register-based study of Danish patients aged ≥65 years included all changes within the analysis period and did not include circumstances for general practice changes. However, there are studies that have attempted to explore circumstances for change of physician.^25,27^ Mold *et al* studied reasons for change of GP in older patients. Most common was involuntary reasons such as doctor dying, retiring, or otherwise leaving practice. Other common circumstances were owing to insurance or cost and patient moving. Relatively few patients (5%) listed dissatisfaction with the doctor as a reason for change and of those who did most had relations lasting 2–9 years.^25^ Nagraj *et al* studied whether voluntary change of GP in the entire population could be used as a quality indicator. They found limited utility of voluntary change as a quality indicator owing to substantial unmeasured variation in disenrollment rates. The strongest associations with change of GP were doctor–patient communication scores, confidence and trust in the doctor, and overall patient satisfaction.^27^

Unexpectedly, the present study found the number of changes of general practice stable across age groups of 5-year age intervals for the older population. It was expected that most people entering retirement would stay listed with the same practice until they moved to a nursing home facility or closer to family at a higher age owing to need for home care. Thus, a trend towards more discontinuity for the oldest old was expected. In support of this hypothesis was the introduction of the designated home care GP programme, where it has become commonplace to change GP to the designated home care GP when moving to a nursing home.^28^ The designated home care GP has been associated with a decrease in number of hospital admissions in nursing homes with successful implementation of the designated GP programme.^29^ A possible reason for the stable frequency of changes across age groups might be that end-of life trajectories,^30^ such as terminal illness, organ failure, and frailty, may be distributed somewhat evenly across the included age groups and thus would show no difference in aggregated data. Another possible explanation might be that the oldest old change general practice owing to health-related circumstances and the youngest old change because of other reasons such as moving owing to changing housing preferences in retirement.

Although this study has shown high levels of site-level COC in Danish general practice during the past decade and a half, the future prospects for COC are uncertain. An increasing number of GPs choose to work as locum, employed staff, or part-time instead of buying their own practice. In addition, many rural areas have GP recruitment problems.^31^ In Denmark this has resulted in a situation where general practices in rural areas are being bought by GPs who collaborate with private corporations to run these practices by employing GPs who often work for short-term employment. Alternatively, in rural areas the national authorities (Danish regions) establish temporary general practices or hire private cooperations for short-term services of a general practice. The discussion in Denmark is heated on this matter,^32,33^ especially as these kinds of general practices might deliver less COC compared with traditional general practices.^32,33^ Nevertheless, this might be part of a larger change in the organisation of Danish general practice to come.

### Implications for research and practice

The results regarding site-level COC in Danish general practice measured in this study are reassuring. With the known benefits of COC, especially for the older population where multimorbidity^4^ and polypharmacy^5^ are the most prevalent, the trend of a 9.5 year-long patient–general practice relation is a sign of general practice fulfilling its goal of providing high levels of COC.^14^ As this study included all cases of discontinuity, future research is needed to investigate the reasons and circumstances for changing general practices.

## Data

Data is protected by Statistics Denmark. Application to access these data must be made to Danish Regions and Statistics Denmark in collaboration with a Danish research institution.
